# Dexmedetomidine attenuates P2X4 and NLRP3 expression in the spine of rats with diabetic neuropathic pain^1^

**DOI:** 10.1590/s0102-865020190110000005

**Published:** 2019-12-20

**Authors:** Liu Kang, Huang Yayi, Zhou Fang, Zhao Bo, Xia Zhongyuan

**Affiliations:** I PhD, Department of Anesthesia, Renmin Hospital of Wuhan University, Wuhan, China. Conception and design of the study, acquisition and interpretation of data, manuscript writing.; II PhD, Department of Anesthesia, Renmin Hospital of Wuhan University, Wuhan, China. Acquisition of data, critical revision.; III PhD, Department of Anesthesia, Renmin Hospital of Wuhan University, Wuhan, China. Acquisition of data.; IV PhD, Full Professor, Department of Anesthesia, Renmin Hospital of Wuhan University, Wuhan, China. Design and supervised all phases of the study, critical revision.

**Keywords:** Neuralgia, Dexmedetomidine, Receptors, Purinergic P2X4, NLR Family, Pyrin Domain-Containing 3 Protein, Rats

## Abstract

**Purpose:**

To evaluate the effects of Dexmedetomidine (Dex) on spinal pathology and inflammatory factor in a rat model of Diabetic neuropathic pain (DNP).

**Methods:**

The rats were divided into 3 groups (eight in each group): normal group (N group), diabetic neuropathic pain model group (DNP group), and DNP model with dexmedetomidine (Dex group). The rat model of diabetes was established with intraperitoneal streptozotocin (STZ) injections. Nerve cell ultrastructure was evaluated with transmission electron microscopy (TEM). The mechanical withdrawal threshold (MWT) and motor nerve conduction velocity (MNCV) tests documented that DNP rat model was characterized by a decreased pain threshold and nerve conduction velocity.

**Results:**

Dex restored the phenotype of neurocytes, reduced the extent of demyelination and improved MWT and MNCV of DNP-treated rats (P=0.01, P=0.038, respectively). The expression of three pain-and inflammation-associated factors (P2X4, NLRP3, and IL-IP) was significantly upregulated at the protein level in DNP rats, and this change was reversed by Dex administration (P=0.0022, P=0.0092, P=0.0028, respectively).

**Conclusion:**

The P2X4/NLRP3 signaling pathway is implicated in the development and presence of DNP in vivo, and Dex protects from this disorder.

## Introduction

Diabetic neuropathic pain (DNP) is one of the most frequent chronic complications of diabetes. It is characterized by spontaneous and induced pain, allodynia, and hyperpathia, which result in a decrease in the quality of life of patients affected by DNP^1^ ^,^ ^2^. However, the pathologic changes responsible for DNP are not completely understood. Therefore, identification of the molecular mechanisms implicated in DNP pathogenesis would promote the development of effective therapies, reducing complications of diabetes, as well as offering enhanced quality of life for patients with DNP.

The development of DNP is dependent on two critical pathogenic factors: the metabolic factor and the vascular factor. The primary metabolic mechanisms include hyperglycemia-activated polyhydric alcohol pathway, accumulation of sorbitol and fructose, inositol reduction, non-enzymatic protein glycosylation, and oxidative stress^3^ ^,^ ^4^. The vascular mechanism is the consequence of diabetes-induced systemic microvascular damage; it leads to energy and nutrient deficiency, inducing a hypoxic and ischemic injury of neural cells. Thus, the development of DNP is a complex process, which engenders the deregulation of the cellular environment, affects energy and nutrient supply for the nervous system, and negatively impacts on metabolic pathways, redox state, and osmotic pressure^5^. Deranged nerve cell microenvironment induces adaptive changes in the nervous system, and contributes to irreversible phenotypic, structural, and functional damage of neurons, providing permissive conditions for the development of DNP.

P2X4 receptor is a ligand-gated non-selective cation channel receptor for endogenous ATP. It is widely distributed on activated microglial cells in the rat brain, spinal cord, and ganglion^6^ ^–^ ^8^. The expression of P2X4 receptors in activated microglial cells is essential for the induction of allodynia. However, the function and the signal transduction pathway mediated by activated P2X4 receptor in DNP remains to be elucidated.

The P2X4 receptor and downstream proinflammatory cytokines are involved in pain-associated signaling pathways in gliocytes, thus triggering neuropathic pain. Therefore, a hypothesis can be raised that an intrinsic connection between them is present. The NLRP3 inflammasome, which is activated by danger-associated molecular patterns (DAMPs) downstream of P2X4, can stimulate the maturation and secretion of cytokines, resulting in cell and tissue injury^7^ ^,^ ^9^. Therefore, P2X4 may regulate proinflammatory cytokine release—such as IL-1β—by mediating endogenous DAMPs and subsequently activating NLRP3 inflammasome.

Dexmedetomidine (Dex) is a newly-introduced highly selective agonist of α2-adrenergic receptors, capable of reducing the tension of sympathetic nervous system and providing an anxiolytic effect^10^. In addition, Dex acts as an analgesic and has a limited suppressive effect on breathing^11^ ^,^ ^12^. Recent studies have shown that Dex can alleviate spinal nerve injury via regulating P2X4. However, its effects on DNP and NLRP3 inflammasome are unknown.

Here, we explored a rat model of DNP to examine the impact of P2X4/NLRP3 signaling pathway on the development and presence of DNP. The protective effect of Dex against DNP was also determined. The objective of this investigation was to elucidate the mechanism of DNP pathogenesis as well as identify novel strategies for its prevention and treatment.

## Methods

All procedures were approved by the Wuhan University Renmin Hospital Animal Ethics Committee.

Male Sprague-Dawley (SD) specific pathogen-free (SPF) rats, weighing 200-220 g with 6-8 weeks of age were purchased from Hunan SJA Laboratory Animal Co., Ltd., Hunan, China. The rats were acclimated individually in transparent plastic boxes at a constant room temperature of 25°C for 1 week.

### Establishment of the DNP model

The rats were allocated randomly into three groups (eight in each group): normal group (N group), diabetic neuropathic pain model group (DNP group), and DNP model with dexmedetomidine (Dex group).

Diabetes in the DNP and Dex groups was induced by intraperitoneal injection with 60 mg/kg of 1% streptozotocin (diluting in the citrate buffer, whose pH was from 4.2 to 4.5). Injection of normal citrate buffer was performed in the N group. Rats with caudal vein blood fasting glucose levels higher than 16.7 mmol/L at day 3 post-injection were considered to develop type I diabetes. Blood glucose and weight of rats was monitored daily for 6 weeks post-injection. Rats in the Dex group received daily intraperitoneal Dex injections (50 μg/kg) for 35 days, beginning at day 8 after streptozotocin administration.

### Reagents

Pentobarbital sodium, hematoxylin, and streptozotocin were obtained from Sigma Co., Ltd. (USA). Strept Avidin-Biotin Complex kit and DAB staining kit were purchased from Wuhan Boster Co., Ltd. (China). Dex was purchased from Hengrui Medicine Co., Ltd. (China). Anti-P2X4 antibody, anti-NLRP3 antibody, and anti-IL-1β antibody were obtained from Cell Signaling Technology (USA).

### Behavioral tests

Mechanical withdrawal threshold (MWT) was tested to evaluate the palmar response to mechanical stimuli. To this end, rats were transferred to a Plexiglas chamber and von Frey filaments were used to apply stimulation to the plantar surface of the left hind paw for 2s. The stimulus intensities were applied at a rate of 2.5 g/s until the animal withdrew its paw. Brisk withdraw or paw flinching was considered as positive response and the force inducing reliable withdrawals was recorded as the threshold. Measurements were triplicatedand results from the 50% withdrawal threshold were avergaed. This test was performed at 2, 4, and 6 weeks after streptozotocin injection.

### Determination of motor nerve conduction velocity (MNCV)

Prior to the experiment, rats received a 1.5% solution of thiopentone sodium (30 mg/kg, i.p.) to induce anesthesia. MNCV was assessed through application of a single 3V stimulus to the sciatic (proximal to sciatic notch) and tibial (distally to ankle) nerves with bipolar needle electrodes. Single square-wave pulses of 0.1 ms duration were delivered. After single stimulus, the compound muscle action potential was recorded from the first interosseous muscle of the hind-paw by unipolar pin electrodes. The recording was a typical biphasic response with an initial M-wave and latency was measured from initial onset to maximum negative peak^13^. During the measurements, the animals’ body temperatures were continually surveilled utilizing a rectal probe digital thermometer, and kept at 37°C with a heating pad. MNCV responses were recorded from the plantar muscles using data acquisition system (AD Instrument Pvt. Ltd., LabChart 7.3, Australia). The MNCV was calculated according to previously described method, using the following formula: MNCV= (*Distance between sciatic and tibial stimulation point*)/(*Latency for sciatic-latency for tibial*)^14^. At the end of the experiment, all animals were euthanized by anesthetic overdose.

### Evaluation of spinal pathology by Nissl stain

Formalin-fixed, paraffin-embedded sections of the spine were dewaxed, rehydrated, and microwaved for antigen retrieval. After cooling and washing with PBS, the samples were treated with 3% hydrogen peroxide in methanol for 10 min to endogenous peroxidase. Then, they were incubated with 10% normal goat serum in PBS for 30 min. Subsequently, Nissl Stain buffer was applied for 10 min with constant agitation, and the sections were washed with double-distilled H_2_O and gradient ethyl alcohol. Finally, the sections were destained with 100% ethyl alcohol and observed under a microscope for cell structure and synaptic morphology.

### Transmission electron microscopy (TEM) of sural nerve ultrastructure

The ultrastructure of the sural nerve was examined by TEM. Briefly, the tissue was fixed with 2.5% (v/v) glutaraldehyde and post-fixed with 1% (v/v) osmium tetroxide, dehydrated, and embedded in epoxy resin. Ultrathin sections were stained with 2% (w/v) uranyl acetate and lead citrate, before evaluation under a Hitachi H-7500 TEM. The density of unmyelinated fibers and Schwann cell nuclei were used to quantify histological parameters through determining the area of axons and the area of fibers including myelin sheaths.

### Extraction of total protein from brain tissue

Before sacrifice, the rats were injected with intraperitoneal 10% chloral hydrate (300 mg/kg) to induce anesthesia. Brain tissue was removed and preserved at −80°C for future utiization. One hundred mg of brain tissue were homogenized and lysed with 10 ml RIPA lysis buffer (100 μl PMSF supplemented with complete protease inhibitors cocktail). The debris was removed by centrifugation and protein concentration in the supernatant was determined by BCA kit.

### Western blot

Cellular lysate was obtained with a RIPA lysis buffer containing 1% Triton X-100 and a complete protease inhibitors cocktail. After incubation for 40 min at 4°C, insoluble components were removed with 15-min centrifugation at 12.000 g. Next, proteins were separated by SDS-PAGE and transferred onto the Immobilon polyvinylidene diﬂuoride (PVDF) membranes (Millipore Corporation, Billerica, MA, USA). These were blocked with 5% skim milk powder and 0.5% Tween 20 in PBS for 2 h at 4°C, and proteins were detected using appropriate antibodies. Horseradish peroxidase-conjugated anti-rabbit or anti-mouse IgG were used as secondary antibodies. Proteins were visualized by chemiluminescence detection reagents before assessment with an Image-Pro Plus 6.0 image analysis system.

### Statistical analysis

Data were analyzed using SPSS 17.0 software and presented as mean ± SD. Statistical significances of the data between the three groups were evaluated by one-way ANOVA test followed by Student-Newman-Keuls *post hoc* test. Differences were rendered statistically significant when *P* < 0.05^15^ ^–^ ^17^.

## Results

### Spinal pathology

**Figure 1:**
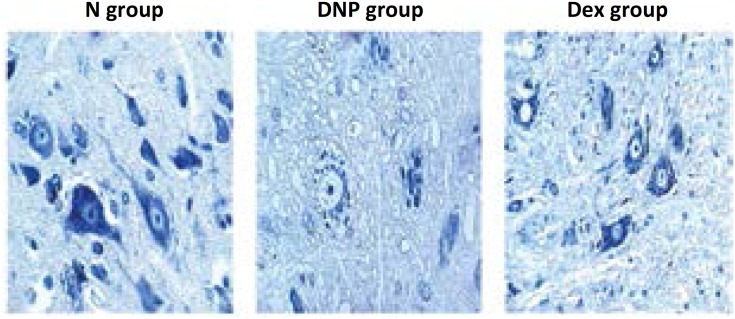
Nissl stain for rat neurocyte in the three experimental groups (×400).

Spinal Nissl stain has shown that neurocytes in the N group were arranged uniformly. The well-organized structure of cytoplasm, nucleus, and organelles could be clearly seen. In comparison with the N group, neurocytes in the DNP group displayed smaller size and lower synapse quantity, and a less compact arrangement. Administration of Dex partially restored the phenotype of neurocytes, but shrunken cells and focal edema continued to be present (Fig. 1).

### Sural nerve structure

**Figure 2:**
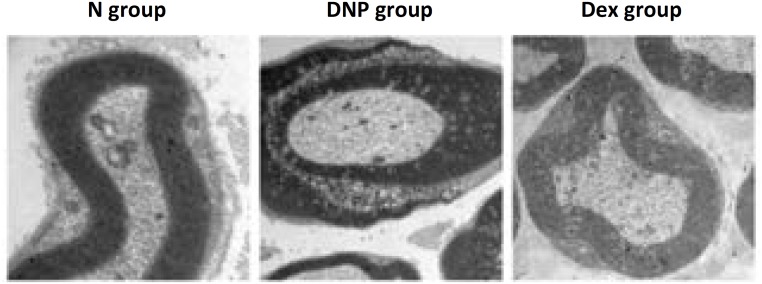
Sural nerve structure assessed by TEM.

Observation by TEM revealed that myelin sheath was arranged regularly in the N group. Mitochondrias with normal morphology were found in axons. Demyelination was commonly found in the DNP group; reduced number of myelinated fibers, disordered arrangement of the myelin sheath, denatured axons, and proliferated Schwann cells were frequently observed. Importantly, the extent of demyelination was clearly reduced in the Dex group, and only a portion of the myelin sheath was disorderly arranged (Fig. 2).

### MWT and MNCV

**Figure 3:**
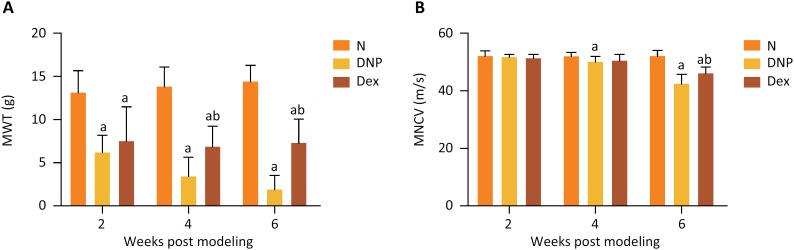
Comparison of MWT (A) and MNCV (B) among the three experimental groups. ^a^ Indicates *P* < 0.05 *vs.* the N group; ^b^ indicates *P* < 0.05 *vs.* the DNP group.

In comparison with the N group, the MWT threshold values of the DNP and Dex groups were evidently decreased at 2 weeks after streptozotocin injection (*P*=0.0002; *P*=0.0021), while the MNCV threshold value was significantly reduced after 6 weeks (*P*=0.0001; *P*=0.0005). In comparison with the DNP groups, the MWT of the Dex group was markedly increased at 4 weeks after induction of diabetes (*P*=0.013), while MNCV was enhanced at 6 weeks (*P*=0.044) (Fig. 3).

### P2X4, NLRP3, and IL-1β expression

**Figure 4:**
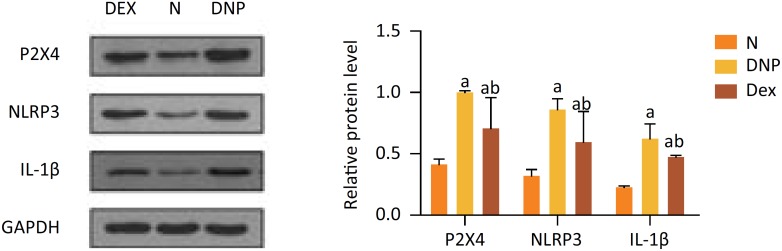
Comparison of P2X4, NLRP3, IL-1β expression in the three experimental groups. ^a^ Indicates *P* < 0.05 *vs.* the N group; ^b^ indicates *P* < 0.05 *vs.* the DNP group.

Western blot analysis documented P2X4, NLRP3, and IL-1β protein expression to be significantly upregulated in the DNP groups (*P*=0.001, *P*=0.0001, *P*=0.0001, respectively) and Dex groups (*P*=0.0014, *P*=0.0030, *P*=0.0001, respectively) as compared to the N group. Moreover, the expression of these three proteins was significantly decreased in Dex-treated rats in comparison with the DNP group (*P*=0.0015, *P=*0.0058, *P*=0.0018, respectively) (Fig. 4).

## Discussion

In this study, we have evaluated the beneficial effects of Dex on DNP in streptozotocin treated rats. We found Dex restored impaired spinal pathology and improved sural nerve structure. In addition, Dex was found to reduce mechanical hyperalgesia and improve the delay of sciatic nerve conduction velocities. Further studies showed that Dex was able to reduce the production of P2X4, IL-1β and NLRP3 levels indicating that Dex holds the ability to protect nerves against inflammatory damage.

Hyperglycemia activates spinal glial cells, causing neuropathic pain, and consequently reduction in MWT and MNCV due to the release of proinflammatory cytokines^18^. Studies have shown that injection of streptozotocin can target and kill the pancreatic beta islet cells and induce type I diabetes in rats^19^. Here, we have induced DNP in rats with intraperitoneal injection of streptozotocin, confirmed with Nissl staining and TEM observations of the sural nerve. Successful generation of the model was further documented by the finding of significantly different MWT and MNDV measurements between normal rats and those with DNP. Impairment of spinal neurocytes is one of the major factors in diabetic neuropathic. Our present study showed a less compact arrangement, smaller size and lower synapse quantity of neurocytes in the diabetic rats. We have found that MWT and MNCV indexes decreased continuously during the development of diabetes in the DNP rat group. However, both parameters were restored after Dex administration. It suggested that Dex could alleviate abnormal sensation caused by diabetes and increase motor nerve conduction velocities.

Previous studies demonstrate that Dex exhibit potential *in vitro* and *in vivo* anti-inflammatory activity^20^ ^,^ ^21^, suggesting its potential in ameliorating inflammation induced by diabetes. Inflammatory cytokines play an important role in neuronal damage, especially in painful neuropathy. The high levels of P2X4 and NLRP3 in STZ treated rats suggested that cytokines are involved in neuropathy^22^. The mechanism of high glucose-induced P2X4 expression is not yet fully understood. It has been reported that hyperglycemic stimulation can activate JAK/STAT signaling pathways by increasing the activity of transcription factor STAT1, thereby increasing the expression of P2X4^23^. Numerous evidence suggests that NLRP3 inflammasome activation may regulate the inflammation process of diabetes mellitus and related complications. It has been documented that P2X4 receptors are involved in DNP through activation of DAMPs like the NLRP3 inflammasome and in the process of NLRP3 protein expression. In addition, P2X4 receptors were capable of mediating the secretion of proinflammatory cytokine IL-1β^24^. Of relevance, in our study the expression of P2X4 and NLRP3 in DNP rats was markedly reduced after treatment with Dex. This finding is consistent with the involvement of the P2X4 receptor in DNP development. The finding of NLRP3 and IL-1β upregulation suggests they intervene in inflammatory factor activation. Administration of Dex led to decreased expression of P2X4, NLRP3, and IL-1β, indicating that Dex suppresses the expression of NLRP3 and proinflammatory factor IL-1β by inactivating P2X4 receptor.

## Conclusions

The administration of the Dex to DNP model rats restored the neuropathy and improved the pathological development of disease. The present results showed that inflammatory factors mediated by upregulation of P2X4 and NLRP3 are responsible for the development of DNP in the rat model, and Dex alleviated the progress by inhibiting the P2X4/NLRP3 signaling pathway and suppressing the inflammatory response.
